# A Simple Method for Estimating Informative Node Age Priors for the Fossil Calibration of Molecular Divergence Time Analyses

**DOI:** 10.1371/journal.pone.0066245

**Published:** 2013-06-05

**Authors:** Michael D. Nowak, Andrew B. Smith, Carl Simpson, Derrick J. Zwickl

**Affiliations:** 1 Institute of Systematic Botany, University of Zürich, Zürich, Switzerland; 2 Department of Palaeontology, The Natural History Museum, London, United Kingdom; 3 Museum für Naturkunde der Humboldt-Universität zu Berlin, Berlin, Germany; 4 Department of Ecology and Evolution, University of Kansas, Lawrence, Kansas, United States of America; Field Museum of Natural History, United States of America

## Abstract

Molecular divergence time analyses often rely on the age of fossil lineages to calibrate node age estimates. Most divergence time analyses are now performed in a Bayesian framework, where fossil calibrations are incorporated as parametric prior probabilities on node ages. It is widely accepted that an ideal parameterization of such node age prior probabilities should be based on a comprehensive analysis of the fossil record of the clade of interest, but there is currently no generally applicable approach for calculating such informative priors. We provide here a simple and easily implemented method that employs fossil data to estimate the likely amount of missing history prior to the oldest fossil occurrence of a clade, which can be used to fit an informative parametric prior probability distribution on a node age. Specifically, our method uses the extant diversity and the stratigraphic distribution of fossil lineages confidently assigned to a clade to fit a branching model of lineage diversification. Conditioning this on a simple model of fossil preservation, we estimate the likely amount of missing history prior to the oldest fossil occurrence of a clade. The likelihood surface of missing history can then be translated into a parametric prior probability distribution on the age of the clade of interest. We show that the method performs well with simulated fossil distribution data, but that the likelihood surface of missing history can at times be too complex for the distribution-fitting algorithm employed by our software tool. An empirical example of the application of our method is performed to estimate echinoid node ages. A simulation-based sensitivity analysis using the echinoid data set shows that node age prior distributions estimated under poor preservation rates are significantly less informative than those estimated under high preservation rates.

## Introduction

The increasingly popular integration of molecular systematics and paleobiology known as molecular divergence time estimation involves estimating the age of extant lineages through analysis of DNA sequence divergence calibrated with data from the fossil record. While estimates of lineage age were historically the purview of paleobiology alone, the widespread development of molecular divergence time estimation methods has fueled the rapid expansion of systematic biology into dating clade ages. Molecular divergence time estimation is fundamentally based on the translation of genetic divergence between taxa into an estimate of the age of their most recent common ancestor (MRCA). Implicitly, this involves calibrating the absolute rate of molecular evolution on a phylogenetic tree, and all methods of molecular divergence time estimation require some externally derived temporal data to provide this calibration [1]. Generally, temporal data from the fossil record of the focal clade or a closely related clade are often employed to calibrate the rate of molecular evolution [2]–[4]. Alternatively, several divergence time studies have employed assumptions of biogeographic history to calibrate node ages, such as the maximum age of a volcanic island [5], [6].

While molecular divergence time estimation is heavily reliant on paleobiological data, the manner in which fossil data are employed as temporal calibrations has consistently generated criticism of divergence time estimates and the conclusions drawn from these estimates [7]–[9]. Much of this criticism specifically cites the misrepresentation of potential sources of error associated with molecular divergence time estimates (see for example [10]). Generally, there are three primary sources of error in divergence time estimates: 1) uncertainty in topology and branch length estimates; 2) uncertainty in the extent of heterogeneity in the rate of molecular evolution; 3) uncertainty in the temporal calibrations provided by fossil data [11]–[16]. Error associated with estimates of topology/branch length and rate heterogeneity (i.e. points 1 and 2 above) have been largely accommodated through the development of Bayesian methods that jointly estimate topology and divergence times and employ sophisticated relaxed-clock models of molecular rate variation such as BEAST [13], [17], MCMCTREE [18], MultiDivTime [19], PhyloBayes [20], and TimeTree [21]. Despite these powerful methodological improvements, a number of recent simulation and empirical studies have shown that the phylogenetic placement and temporal representation of fossil calibrations (i.e. point 3 above) represent the most significant contribution to imprecision and potential inaccuracy in node age estimates [9], [11], [12], [14], [15], [22]–[24].

It has long been appreciated that the age of the oldest fossil taxon confidently attributable to a given clade represents the minimum age for the MRCA of that clade and its sister clade [2]. While these minimum age constraints can be easily and relatively confidently interpreted from the fossil record, all methods of molecular divergence time estimation (e.g. Bayesian, likelihood, etc.) require some information to calibrate the maximum age of the clade. Aside from a few well-documented paleobiological events and/or fossil lineages that can be applied confidently as maximum age constraints (see for example [3], [25]), maximum ages are notoriously difficult to interpret from the fossil record [2], [9], [14], [26].

This uncertainty in assignment of maximum age constraints in divergence time analyses has lead to the recent development of Bayesian divergence time methods that allow node ages to be temporally constrained through the application of parametric prior probability distributions, which are meant to reflect the researchers confidence in the temporal calibration provided by the fossil data at hand [13], [20], [21], [27]. Node age priors in Bayesian divergence time analyses can be theoretically expressed using any statistical distribution, but the most common are uniform, exponential, lognormal, gamma, normal, or truncated normal distributions because these tend to represent diminishing probability at greater ages (i.e. “soft-bounds”, see [16], [27]). While these methodological advances provide the framework for integrating uncertainty in temporal calibrations provided by fossils, they do not provide an explicit means of quantifying this uncertainty. It follows then that many of the studies that have employed such prior probability distributions to calibrate node ages have employed somewhat arbitrary parameterizations of these node age priors (see for example [28], [29]). Ho and Phillips [1] among others have suggested an alternative solution by applying “soft-bound” node age priors whose 95% densities are based on well-reasoned arguments from the paleobiological literature (see for example [30]), but such arguments tend to be idiosyncratic and subjective. This is particularly relevant because it has been shown by several authors now that the parameterization (i.e. shape) of node age prior distributions can significantly impact the resulting node age estimates [15], [31].

It is clear now that there is a need for objective means of informing the construction of parametric node age prior distributions based on analyses of fossil data [26]. Marshall [32] has proposed the use of stratigraphic confidence intervals as a means of constructing biologically meaningful prior distributions. Marshall's [32] method requires a fixed topology with branch lengths proportional to relative time (i.e. an ultrametric tree), and represents an elegant and computationally simple means of estimating a potentially informative node age prior distribution. Despite this, the input requirements of Marshall's method limit its practical application for Bayesian divergence time analyses, as fossil calibrations act as prior distributions to inform the estimation of an ultrametric tree and thus these processes are not easily decoupled [33]. More recently, Wilkinson et al. [34] developed a method for constructing a node age prior distribution based on an analysis of the primate fossil record. Their method employs a stochastic forward-modeling approach to simultaneously estimate parameters of the diversification process (i.e. speciation and extinction) and process of fossil preservation. While their method is both elegant and powerful, it is also quite complex and appears to be tailored to the primate fossil record, and thus it may be difficult to apply to other taxonomic groups [9], [35].

**Figure 1:**
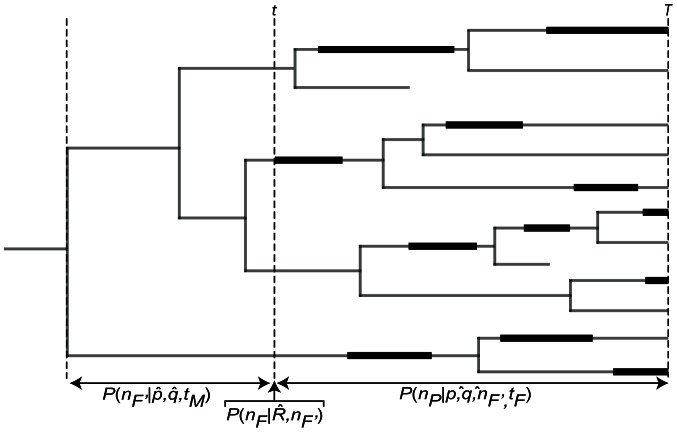
Simplified Diagram of the Model. Our method provides an estimate for the length of time after age of the MRCA of a clade but prior to the age of the oldest fossil (i.e. the missing history). This hypothetical clade has N  =  11 lineages at time T, representing the current standing diversity of the group. Thick bars on the internal branches of the tree represent the preserved fossil history of the clade, such that n  =  1 lineage preserved at time t. The expressions for deriving the probability of the three key temporal durations in the history of a clade are shown.

We present here an alternative approach to constructing informative prior distributions on node ages for use in Bayesian divergence time estimation software packages. Our method fits a branching model to paleobiological data relating the stratigraphic range of all fossil taxa that can be confidently assigned to a given clade in the extant phylogeny of a group to estimate the age of the most recent common ancestor (MRCA) of that clade. The difference between the age of the MRCA and the oldest fossil assignable to a clade represents the amount of time that passed after the clade's origin but before the first recovered fossilization event (Figure 1), and is referred to as the “missing history” of a clade [36], [37]. Briefly, the method we describe utilizes the entire fossil history of a clade (i.e. the stratigraphic ranges of all relevant fossil lineages) to fit a model of lineage diversification, which is then used together with a user-supplied estimate of the per-interval fossil preservation and sampling probability (herein referred to as the “fossil preservation rate”) to estimate the amount of missing history before the first preserved fossil attributable to the focal clade. The primary output of this approach is a probability distribution of the missing history estimate for a given clade, which can then be summarized by fitting a simple parametric probability distribution for use as a node age prior in a Bayesian divergence time program such as BEAST [13]. While in many ways similar to the approach developed by Wilkinson et al. [34], our method differs in that it was specifically designed to be easily applied to nearly any clade with a reasonably diverse fossil record (i.e. sufficient to estimate the key origination and extinction rate parameters based on the distribution of fossil lineages in a clade). Relying on a few key assumptions regarding the diversification history of the focal clade, our method achieves an ease of application that makes it a powerful addition to the suite of available approaches for assigning node age priors.

The performance of the method is evaluated by analyzing a diverse set of simulated data sets, and an example empirical application of the method is conducted to estimate divergence times in echinoids using fossil distribution data and a DNA sequence dataset from Smith et al. [38]. Echinoid divergence time estimates based on informed priors are compared to estimates generated with priors established through the “traditional” application of fossil-based minimum age constraints. While our method was designed to estimate a node age prior for a single clade (i.e. a node in an extant phylogeny), we explain how the method can be applied iteratively throughout a given phylogeny when more than a single clade has a suitably diverse fossil record. The calibration scheme we employ in estimating echinoid divergence times provides an example of such an iterative application of the method to generate several informative priors for sub-clades, which we subsequently apply in concert to constrain a molecular divergence time analysis using the BEAST software package [13], [17]. Finally, a second simulation study on the echinoid fossil range data is performed to examine how the precision of priors estimated by our method is affected by varying rates of fossil preservation and recovery.

## Materials and Methods

The method we describe here employs the logical framework developed by Foote et al. [36], who analyzed the mammal fossil record using a conditioned model of diversification (i.e. birth-death branching model) and fossil preservation to estimate the age of the MRCA of extant mammals. Their analysis was specifically designed to independently evaluate the remarkable discrepancy between estimates of the age of mammal origins based on molecular clock studies and estimates based on the fossil record. While Foote et al. [36] was focused on hypothesis testing, we have modified their model to estimate the amount of time prior to the oldest fossil in a clade directly from parameters of the fossil record (see Figure 1). The method described below estimates the “missing history” of the fossil record for a clade of interest by fitting the observed stratigraphic distribution of fossil lineages attributable to that clade to a model of cladogenesis conditioned on known clade diversity at both the extant time (i.e. current standing diversity) and within the clade's oldest stratigraphic interval. The accuracy of the method is contingent upon accurate counts for fossil lineage diversity in the oldest stratigraphic bin of the clade of interest, and thus the effects of incomplete fossil preservation are incorporated through model averaging.

### Data

Our method requires as input the stratigraphic range of each fossil taxon confidently assignable to a given clade (e.g. first and last occurrence at minimum), an estimate of the current standing diversity, and an estimate of the fossil preservation and recovery rate for the fossil record of the clade of interest. While no specific phylogeny is required or utilized by the method, as with any temporal calibration based on fossil data, one should be confident that the clade of interest represents a natural and monophyletic group. The stratigraphic data associated with each fossil taxon must be expressed in terms of a consistent stratigraphic binning scheme (e.g. see Table S1). The use of a binning scheme is conventional when dealing with fossil data because the exact age of a geologic formation holding a fossil is often not known, but a range of dates can often be estimated based on comparative analysis with surrounding strata (for review see [4], [39]). As currently configured, the software that implements our method is capable of interpreting stratigraphic ranges expressed in terms of International Stratigraphic Commission (ISC) Stages [39], or PBDB 10 Ma Bins (The Paleobiology Database 2008). While it is theoretically possible to employ any binning scheme, it is important to consider the fact that statistical power is increased proportional to the absolute number of stratigraphic bins. Therefore, a binning scheme with higher resolution should provide better parameter estimates, with the caveat that the fossil occurrence data can be confidently assigned to such bins.

### Estimating Origination and Extinction Rates

Origination and extinction rates (sometimes called “birth” and “death” rates) are estimated directly from fossil stratigraphic range data using methods developed by Foote [40]. Briefly, Foote's method provides per-capita estimates of origination and extinction rates for a single stratigraphic bin. There are four fundamental classes of lineages for a given bin: 1) those that cross only the lower boundary of the bin (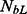); 2) those that cross only the upper boundary of the bin (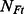); 3) those that cross both the lower and the upper boundary of the bin (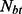); 4) those with a range confined to the bin (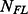). The sum of 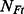 and 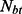 provides the total number of lineages crossing the upper boundary (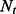), and the sum of 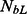 and 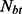 provides the total number of lineages crossing the lower boundary (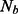). The per-capita rates of origination (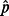) and extinction (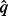) are given by the equations
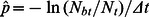


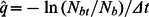



where 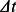 is the temporal length of the bin in question. In this way, origination and extinction rates are estimated for each bin throughout the preserved stratigraphic distribution of the clade. For any given clade estimates of origination and extinction rate can vary considerably through time. While such fluctuations may be pertinent to the diversification history of the clade, it can be difficult to decouple the signal of such processes from that of preservational anomalies [41], [42]. The model of cladogenesis we employ assumes constant diversification rates through time, and subsequently the estimated average rates of origination (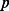) and extinction (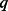) are used in all equations herein.

### The Model

The core of our method is based on the branching process derivations originally performed by Raup [43] and Foote et al. [36]. We provide here a brief summary of the model that serves as the logical framework for the method, but a more detailed derivation can be found in Foote et al. [36] and Raup [43]. The primary formula is the probability of starting a stratigraphic interval of length 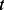 with 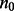 lineages and ending with 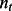 lineages, 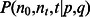 (i.e. this is equivalent to 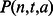 in [36]). A special case of this is the probability of complete extinction (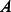), which is given by
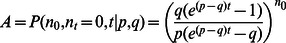



By subtraction, the probability of survival over the interval is then given by
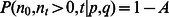



Another special case of particular interest is starting with one lineage (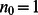) and ending with 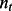 lineages, given by
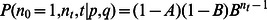



where again 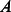 is the probability of extinction (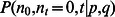, see above), and 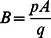



Similarly, for other values of 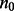 and 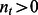


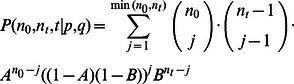



In our case, we have two time intervals of interest: the length of the interval from the MRCA to the first known fossil occurrence (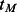, i.e. the “missing interval” of [36]), and the observed time interval from the first known fossil occurrence to the present time (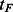). We also have three variables representing the diversity of the clade of interest: the number of extant lineages known to exist at the present time (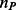); the observed number of fossil lineages in the oldest stratigraphic bin of the clade of interest (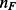); an estimate of the true number of fossil lineages in the oldest stratigraphic bin (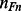).

Our method requires the user to supply an estimate of the per-interval fossil preservation and sampling probability (i.e. preservation rate) of the clade of interest (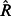) in order to estimate the true diversity of the clade in the first stratigraphic bin (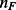) based on the diversity of the clade at this time observed from the fossil record (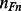). This relationship is modeled by a binomial distribution with probability mass function 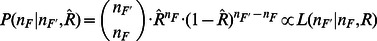



Our method implicitly relies on the assumption that 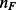 (i.e. the true diversity in the first bin) is less than the assumed known extant diversity of the clade (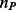). For some taxonomic groups (e.g. clades showing explosive radiations shortly after their first appearance) this assumption will be violated, and in such cases it would not be appropriate to use this method.

At this point the data (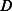; the fixed values) are 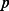, 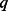, 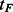, 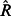, 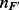, and 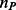, and the unknowns are 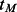 (the missing interval) and 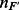 (a nuisance parameter that we ideally want to integrate out). If we ignore estimating 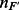 for the moment, the likelihood is 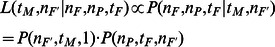



or the probability of starting with one lineage and ending with 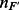 lineages over the missing interval of length 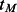 times the probability of starting with 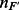 lineages and ending with 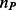 lineages over the interval 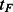. We now can get the total likelihood of 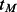
by summing over values of 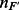, stopping at some arbitrary point when additional terms contribute little to the likelihood.
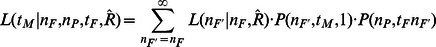



**Figure 2:**
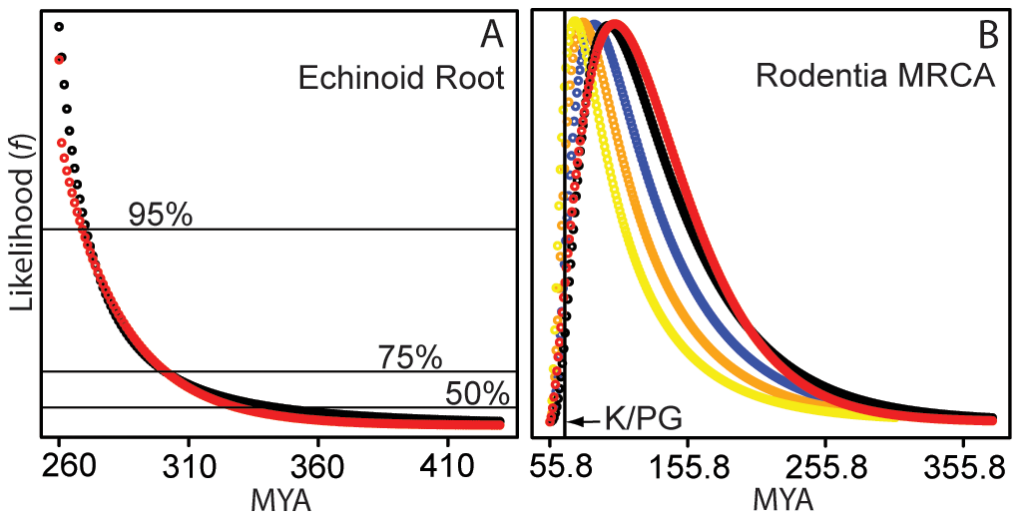
Example informative divergence time priors estimated with the SNAPE v1.0 software. These likelihood curves and associated best-fit gamma distributions show some of the variation in prior shape that can be estimated using this method. The y-axis scale is the likelihood (or *f* for the best-fit gamma distribution) and the x-axis is in millions of years ago (MYA). Note that the scale of discretized likelihood curve and the gamma distribution are not equivalent, and they must be scaled to assist in visualization. **A**. Estimated prior distribution for the root node in the echinoid data set. Values of the discretized likelihood curve are shown in black, and the best-fit gamma distribution is shown in red. Horizontal lines representing the 95%, 75%, and 50% quantiles of the discretized likelihood curve are labeled on the figure. The quantile values are shown here only for reference when interpreting the simulation results shown in Figure 3. **B**. Estimated prior distribution for the MRCA of the mammalian order Rodentia. The input data for this prior estimate was assembled by searching the Paleobiology Database (www.pbdb.org) for all Rodentia occurrences (see File S2). This analysis assumed the existence of 400 extant genera in Rodentia. The oldest Rodentia fossil occurrence that met the input data criteria was 55.8 Ma. The vertical line shows the position of the Cretaceous/Paleogene (K/PG) boundary at 65.5 Ma. The analysis was performed once for each of four preservation rates: 0.1  =  black; 0.2  =  blue; 0.3  =  orange; 0.4  =  yellow. The best-fit gamma distribution for the likelihood curve assuming a 0.1 preservation rate is shown in red. This prior for the age of the MRCA of Rodentia was estimated solely for demonstration purposes. The results show how the preservation rate estimate provided by the user can have a large impact on the shape of the prior estimated.

We implicitly assume a uniform prior on 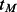 and fit a parametric distribution to the discretized likelihood curve (see below), which is used as a prior on 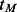 in molecular divergence time analyses. A graphical representation of the problem is shown in Figure 1 (but also see [34], and Figure 2 shows two example discretized likelihood surfaces).

### Fitting a Parametric Probability Distribution to the Discretized Likelihood Curve of 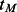


The primary output from the method described above is a list of likelihood values for missing intervals of time (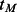) and a proposed zero offset value representing a conservative estimate for the minimum age of the clade of interest. The discrete likelihood curve represented by this list cannot be applied directly as a prior probability distribution for a given clade. Bayesian molecular divergence time software packages (e.g. BEAST, PhyloBayes, TimeTree) require priors to be specified under relatively simple parametric distributions (e.g. uniform, exponential, gamma, lognormal). Thus a specific parametric distribution with appropriate parameter values must be chosen to mimic the discretized likelihood curve generated by the method (e.g. see Figure 2). In the software that has been developed to implement our method, we have attempted to extract the information from the discretized likelihood curve through a least-squares distribution fitting function. Theoretically any parametric distribution could be used to fit the discrete likelihood curve, but we have chosen a gamma distribution for implementation in our software tool because it performed consistently well in fitting a diverse set of likelihood curves tested during software development. Still it is important to note here that the likelihood curves produced for some data sets can be quite complex, and thus fitting a gamma distribution can be very difficult. Therefore, the fit between the discrete likelihood curve generated by the software tool and the parametric distribution to be employed as a node age prior must be visually validated and, when necessary, manually adjusted by the user to accurately characterize the node age prior probability distribution.

The method described above is implemented in an open source software package written in C++ called the Single Node Age Prior Estimator (SNAPE) v1.0 (https://github.com/michaeldnowak/snape).

### Performance of the Method with Simulated Data

To evaluate the performance of the method under various scenarios of diversification and fossil preservation, clades of fossil lineages were simulated and subjected to incomplete preservation. Briefly, a branching model of cladogenesis was employed to simulate a clade that originated 250 million years ago and diversified under an origination rate of 1.0 and an extinction rate of 0.9. The stratigraphic ranges of the resulting fossil lineages were binned according to ISC stages and the effects of incomplete sampling and preservation on the ranges of these lineages were simulated under a specific preservation rate. The branching process simulation was performed for 100 replicates and each replicate was subjected to the simulated fossil preservation process 10 times. The simulation was conducted under three different preservation rates (0.8, 0.45, and 0.1), yielding a total of 1000 replicates for each preservation rate. Using as input the preserved fossil record, the true number of extant lineages of each simulated clade, and the simulated preservation rate, the length of missing history (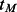) was estimated for each clade and a parametric prior distribution was fit to the resulting likelihood curve using the SNAPE v.1.0 software tool. Simulated data sets were constructed using R scripts (see File S1) and software written in the C programming language by C. Simpson.

### Echinoid Divergence Times

Smith et al. [38] employed a data set consisting of 3680 nucleotides sequenced from one mitochondrial (16 S large subunit) and two nuclear rRNA genes (18 S small subunit, and 28 S large subunit) to reconstruct the phylogeny of extant echinoids and estimate divergence times in the clade. The resulting data set includes representatives of thirteen of the fourteen extant echinoid orders and resulted in approximately 70% coverage of extant echinoid families. The molecular clock was significantly rejected in their study, and thus relaxed-clock models of molecular evolution were applied in a number of different molecular divergence time software packages including multidivtime. Divergence time estimates were then examined for congruence with the observed echinoid fossil record. Molecular and fossil-based estimates of clade age were examined for congruence in a number of focal nodes. Their results show congruence between these independent sources of data in approximately 70% of the nodes tested [38].

The echinoid fossil record is particularly well suited to the estimation of informative divergence time priors due largely to the compilation of detailed stratigraphic range data for all relevant fossil genera in a comprehensive database (The Echinoid Directory; http://www.nhm.ac.uk/research-curation/research/projects/echinoid-directory/). Furthermore, well-preserved morphological synapomorphies allow fossil genera to be confidently placed within clades of the extant echinoid phylogeny [44]. We estimate informative divergence time priors for eight well-supported nodes (i.e. Bayesian posterior probabilities greater than 0.95 and likelihood bootstrap proportions greater than 70%) from the phylogenetic analyses of Smith et al. [38] and apply these as constraints in the estimation of echinoid divergence times in the Bayesian divergence time estimation software BEAST v1.7.2 [13]. Briefly, stratigraphic range data for all fossil echinoid genera were compiled from the Echinoid Directory and used to construct data sets of fossil lineages attributable to the clades defined by the eight constraint nodes (see Table S1). While both the ISC Stages [39] and PBDB 10 Ma Bins (The Paleobiology Database 2008) were found to be suitable stratigraphic binning schemes for these data, we found the ISC Stages favorable due to the increased stratigraphic resolution it allowed. A complete list of fossil echinoid genera used in this study with associated stratigraphic ranges is provided in Table S1. Informative gamma-distributed divergence time priors for each of the eight constraint nodes were estimated through the method described above. A high preservation rate estimate of 0.8 was chosen because echinoids are thought to have relatively high rates of fossil preservation, and while not based explicitly on an analysis of the echinoid fossil record, this value is consistent with independent paleobiological evidence [38], [44]. Furthermore, while we chose to analyze these data under a single preservation rate estimate (0.8), it would be practical to examine a range of potential preservation rates if the goal of this study was primarily aimed at estimating echinoid divergence times, rather than providing an example implementation of the method. A set of uniform divergence time priors for the same eight nodes was also established using the oldest fossil occurrence attributable to each constraint node as a minimum age (see below).

Two sets of BEAST v1.7.2 analyses were performed with identical settings, but differing in the application of constraint node age priors: 1) Uniformly-distributed priors determined through minimum and maximum age constraints; 2) Informative gamma-distributed priors as estimated through the method described above. In the “uniform” set, prior distributions were established such that lower bounds (i.e. minimum age constraints) represent the age of the oldest stratigraphic bin containing an appropriate fossil taxon for a given constraint node, and upper bounds (i.e. maximum age constraints) were set to 355 Ma for all constraint nodes. A maximum age constraint is required when a uniform prior is employed, and our choice of 355 Ma represents an unreasonably old age for the root of the tree based on the absence of any crown-group echinoids in the two previous (e.g. younger) stratigraphic bins. The “gamma” analysis set applied gamma-distributed node age priors estimated through the method described above for each of the eight constraint nodes.

All BEAST analyses were performed in triplicate (i.e. three independent chains), with each chain allowed to run 8 million generations and sampled every 1000 generations to provide an estimate of the posterior distribution. The best-fit substitution model was found to be GTR+G+I through the application of the Akaike Information Criterion (AIC) with the program MrModeltest v2.3 [45]. In BEAST, the default parameterization of the birth/death model of cladogenesis was employed as the tree prior, and the rate of molecular evolution was assumed to vary between branches following a lognormal distribution with default parameters. As suggested by Heled and Drummond [46], a separate single chain was run in which the sequence data was excluded to confirm the absence of anomalous structure in the joint prior distribution (i.e. deviating from expectations given the priors employed (see Figures S2 and S3). In all BEAST analyses the eight constraint nodes were constrained to be monophyletic and each analysis was provided with the same starting tree, which conformed to both to the topological constraints and minimum age constraints as derived from fossil range data for each constraint node.

The program Tracer v1.5 was used to confirm suitable effective sample size of all parameters estimated from the posterior distribution of trees (i.e. ESS greater than 100; [13]). Additionally, Tracer provided visual confirmation of the stationarity of each chain following removal of a suitable burn-in and convergence of the three runs for each analysis set. Based on these results, the BEAST utility program LogCombiner v1.7.2 was used to remove the first 800 trees from the posterior distribution as burn-in, and the remaining trees from the three runs were combined to yield a final posterior distribution of 12600 trees for each of the two analysis sets (i.e. uniform and gamma-distributed priors). The BEAST utility program TreeAnnotator v1.7.2 was used to calculate the posterior probabilities of branches, the posterior distribution of node times, and the maximum *a posteriori* tree, which was then annotated with branch and node posterior summaries and exported in nexus format for visualization in the program FigTree v1.3.1.

### Simulating the Effects of Incomplete Preservation on the Echinoid Fossil Record

An appropriate examination of the effects of incomplete preservation requires raw occurrence data for all fossil taxa. Occurrence data relating to a single fossil taxon represents a global compilation of every published and unpublished observation of that fossil taxon. While such data are ideal for studies of analytical paleobiology, occurrence data have thus far been exhaustively compiled for only a few fossil taxa (but see the Paleobiology Database 2008). Since occurrence data were not immediately available for the echinoid fossil record, we simulated fossil occurrence data within the observed stratigraphic ranges of all fossil echinoid genera. Observed stratigraphic ranges were populated with simulated occurrences in discrete stratigraphic bins following a beta distribution (α  =  β  =  2) between the first and last stratigraphic bins for each generic range. This procedure was designed to mimic the well-documented observation that most fossil ranges are relatively occurrence-poor in the “tails” compared to the rest of the range [47]. Occurrences were added to the observed range of each fossil echinoid genus by sampling from an exponential distribution with a mean of 14. In this way, each echinoid genus had at minimum one (singletons) or two occurrences to define their observed range. The mean of 14 occurrences to add to the observed range was chosen because it corresponds to the mean number of occurrences calculated from all of the fossil echinoid genera in the Paleobiology Database (2008). It was impossible to calculate this value from the Echinoid Directory because this database does not contain stratigraphic data at the level of occurrence. Observed ranges were populated with occurrences 100 times for each constraint node in the echinoid tree. The resulting eight occurrence data sets were subjected to random sub-sampling according to four preservation rates: 0.20, 0.40, 0.60, and 0.80. This generated four occurrence data sets, each consisting of 100 replicates sub-sampled under a single preservation rate. This procedure generates 400 occurrence data sets (i.e. 100 replicates for each of four preservation rates) for each of eight constraint nodes in the echinoid tree. An informative gamma-distributed prior was estimated for each occurrence data set (a total of 3200) using the method described above. The preservation rate provided for the calculation of node age priors was identical to the preservation rate employed to sub-sample the data (i.e. 0.20, 0.40, 0.60, and 0.80). This allowed our study to limit the number of potentially confounding factors that might impact the precision of the priors estimated.

## Results

### Performance of the Method with Simulated Data

**Figure 3:**
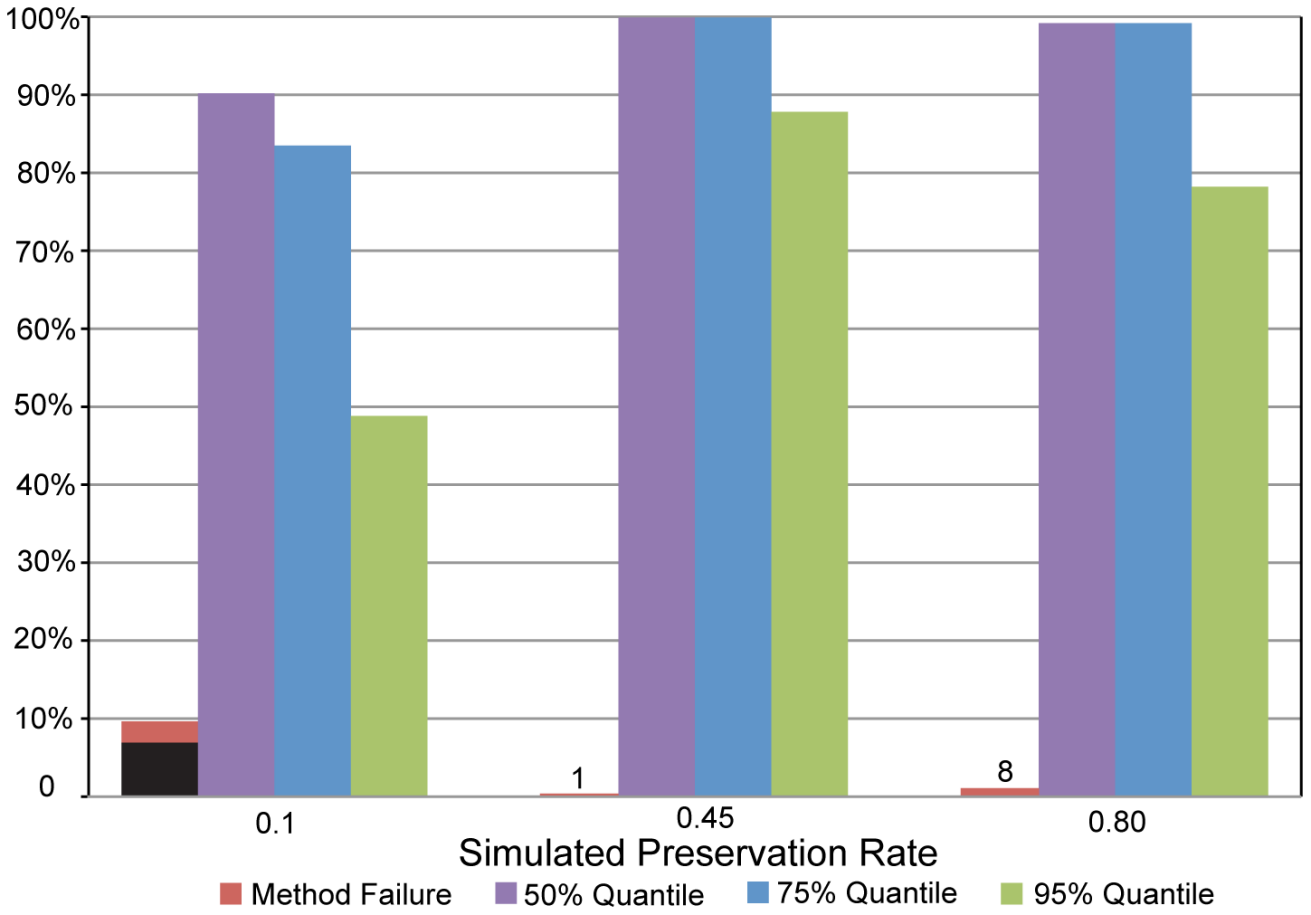
Performance of the method with simulated data. For three different preservation rate categories (0.1, 0.45, and 0.8) a total of 1000 simulation replicates were analyzed using the SNAPE v1.0 software. Method success was determined by the likelihood of the true TMRCA being greater than the 50% quantile of the discretized likelihood curve, which is shown by the purple bars. The percentage of replicates in which the method failed to meet this standard is shown in red. Replicates that failed due to an inability to calculate origination and extinction rates are shown in black. Simulation replicates in which the method returned a prior in which the likelihood of the true TMRCA was greater than the 75% quantile were considered accurate and these are shown in blue. Those replicates in which the prior showed the likelihood of the true TMRCA was greater than the 95% quantile were considered highly accurate, and the proportion of replicates meeting this standard are shown in green.

Simulated data sets were constructed to test the method's capacity for accurately estimating the missing history prior to the oldest simulated fossil occurrence of a clade. The sensitivity of the method to varying preservation rates was evaluated by constructing three unique groups of simulated data sets representing high preservation (0.8), moderate preservation (0.45), and low preservation (0.1). The accuracy of missing history estimates is assessed by evaluating for each simulation replicate if the likelihood of the true age of the TMRCA is greater than the 50%, 75%, or 95% quantile of the discretized likelihood surface (e.g. see Figure 2). We establish a minimum bound for success of the method as those replicates for which the likelihood of the true TMRCA is greater than the 50% quantile of the discretized likelihood curve. We consider simulation replicates for which the likelihood of the true TMRCA is greater than the 75% quantile as accurate, and those that are greater than the 95% quantile as highly accurate. As can be seen in Figure 3, the success rate of our method was very high across all simulation replicates. When the fossil preservation was low (0.1) the method succeeded in 902 out of 1000 replicates, but of the 98 failed replicates 69 were due to an inability to calculate origination and extinction rates because the stratigraphic ranges of the simulated fossil lineages were not sufficiently overlapping. In the low preservation rate data sets the method provided accurate estimates for 83.5% of the replicates, and highly accurate estimates for 48.8% of the replicates. When data sets were simulated under moderate (0.45) or high (0.9) preservation rates the success rate was greater than 99%, and the method produced accurate estimates more than 95% of the time. Highly accurate estimates were produced by the method for 87.8% of the replicates under moderate preservation and for 97.2% of the replicates under high preservation. It is important to note here that it was often difficult to fit a gamma distribution to the discretized likelihood surfaces produced for the simulated data sets. The relatively simple distribution fitting algorithm employed by our SNAPE v1.0 software failed to provide an appropriate gamma distribution for 40.8%, 66.5%, and 1.5% of the replicates simulated under low, moderate, and high preservation rate, respectively.

### Informative Priors Improve the Precision of Echinoid Divergence Times

**Figure 4:**
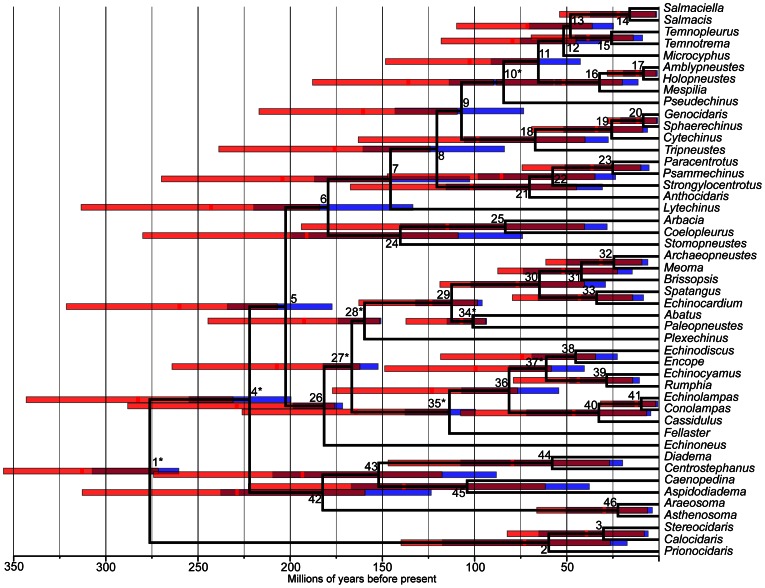
Echinoid divergence times estimated using two alternative node age prior calibration schemes. Bars on nodes represent the 95% HPD of the node age and are colored by the two prior calibration schemes used: red bars  =  uniform priors; blue bars  =  informative gamma priors; purple  =  overlap of 95% HPD from both approaches. The tree represents the highest *a posteriori* chronogram for the analyses run with informative gamma priors, and the nodes are placed at the mean of the posterior distribution of node age. The bright red vertical dash on each node bar represents the mean of that node's age from the posterior distribution of the analyses run with uniform priors. Nodes are numbered as in Table 2, and calibration nodes are indicated with an asterisk. The scale at the bottom of the figure is in millions of years before present (Ma), and the time scale is binned by 50 Ma intervals. The tips of the tree are labeled by genus name as in Smith et al. [21], [38]. Posterior clade probabilities are provided in Figure S1.

Our method was developed to estimate node age priors that are more informative than the standard application of priors reflecting the minimum age of a node implied by the oldest fossil attributable to that clade. To test our method using empirical data, we estimated informative priors for eight constraint nodes and compared divergence time estimates in echinoids with the results of identical analyses using minimum-age priors established through conservative procedures. The resulting divergence time priors employed in the analyses of echinoid node ages (i.e. uniform minimum-age and informative gamma priors) and parameter estimates generated from fossil distribution data of each constraint node (i.e. origination and extinction rates) are shown in Tables 1 and 2. Our estimates of the average origination rate are consistently higher than average extinction rate, suggesting that the clade may not be at equilibrium carrying capacity. The BEAST analyses performed to estimate echinoid divergence times are summarized in the time calibrated ultrametric phylogeny shown in Figure 4, and clade credibility values can be found in Figure S1. A more thorough summary of the node age estimates are shown in Table 2, where it can be seen that the mean divergence times (%D_mean_) estimated through the application of minimum age constraints were on average 27% (i.e. nearly 30 million years; D_mean_) older than those estimated with informative gamma distribution priors calculated with our method. Furthermore, 95% highest posterior densities (HPD) of the posterior distribution of node ages (i.e. a measure of the precision of the posterior estimate;%D_HPD_) were on average 35% (i.e. nearly 33 million years; D_HPD_) larger than those estimated with informative gamma distribution priors.

**Table 1:** Node age priors employed to estimate echinoid divergence times.

	Minimum Age Constraints		Gamma-Distributed Priors				
Node	Uniform Prior	Joint Uniform Prior Mean and 95% Density	Gamma Prior (shape, scale)	Prior 95% Density	Joint Gamma Prior Mean and 95% Density	Origination Rate (*p*)	Extinction Rate (*q*)
1(root)	*U*(260.4, 355)	320 (273.3, 355)	Gamma(0.9773, 25.83979)	(261.6, 336.7)	279.6 (260.4, 319.4)	0.0578	0.0449
4	*U*(199.6, 355)	288.4 (227.8, 345.4)	Gamma(0.9773, 27.39726)	(200.9, 280.5)	226.2 (199.6, 263.5)	0.0573	0.0449
10	*U*(40.4, 355)	144 (45.4, 246.5)	Gamma(0.9910, 11.57407)	(40.9, 74.9)	56.7 (40.4, 83.9)	0.1023	0.0356
27	*U*(171.6, 355)	233.1 (172.1, 296.2)	Gamma(0.9773, 19.60784)	(172.5, 229.5)	184.6 (171.6, 206.1)	0.0756	0.0585
28	*U*(150.8, 355)	190.4 (150.8, 247.7)	Gamma(0.9743, 18.79699)	(151.7, 206.2)	161.7 (150.8, 178.3)	0.0754	0.066
34	*U*(93.5, 355)	123.2 (93.5, 173)	Gamma(0.9802, 25.06266)	(94.7, 167.6)	107 (93.5, 134)	0.0702	0.0534
35	*U*(99.6, 355)	158.1 (99.6, 228.8)	Gamma(0.9734, 14.81481)	(100.3, 143.2)	111.6 (99.6, 134.2)	0.0913	0.1024
37	*U*(40.4, 355)	87.1 (40.4, 153.2)	Gamma(0.9792, 14.70588)	(41.1, 83.9)	52 (40.4, 74.4)	0.0719	0.1012

**Table 2:** Summary of echinoid divergence time estimates comparing uniform and informative gamma priors.

	Uniform Priors	Informative Gamma Priors	Summary			
Node	Mean Node Age (95% HPD)	Mean Node Age (95% HPD)	Dmean	%Dmean	DHPD	%DHPD
**1 (root)**	**317.34 (271, 355)**	**276.16 (260.4, 307.46)**	**41.18**	**12.98%**	**36.94**	**43.98%**
2	73.89 (25.9, 139.35)	59.85 (17.34, 117.63)	14.04	19.00%	13.16	11.60%
3	39.89 (7.43, 81.8)	30.21 (6, 65.29)	9.68	24.27%	15.08	20.28%
**4**	**286.59 (230.42, 342.64)**	**222 (199.6, 254.9)**	**64.59**	**22.54%**	**56.92**	**50.72%**
5	263.72 (206.44, 320.88)	202.46 (177.32, 234.16)	61.26	23.23%	57.6	50.33%
6	246.31 (183.41, 312.85)	179.48 (133.54, 219.88)	66.83	27.13%	43.1	33.30%
7	207.12 (145.5, 269.3)	145.67 (102.73, 186.9)	61.45	29.67%	39.63	32.01%
8	178.36 (123.56, 238.13)	120.54 (83.92, 160.64)	57.82	32.42%	37.85	33.04%
9	162.77 (108.91, 216.33)	107.23 (73.46, 143.27)	55.54	34.12%	37.61	35.01%
**10**	**137.9 (89.25, 187.3)**	**84.3 (57.41, 113.92)**	**53.6**	**38.87%**	**41.54**	**42.37%**
11	104.22 (64.73, 147.81)	65.55 (42.73, 91.19)	38.67	37.10%	34.62	41.67%
12	80.68 (44.38, 117.61)	51.81 (31.22, 75.37)	28.87	35.78%	29.08	39.71%
13	72.39 (35.64, 109.19)	48.17 (24.84, 71.12)	24.22	33.46%	27.27	37.08%
14	22.36 (1.25, 53.37)	15.97 (1.31, 37.36)	6.39	28.58%	16.07	30.83%
15	39.03 (13.39, 68.8)	25.92 (8.98, 45.65)	13.11	33.59%	18.74	33.82%
16	52.57 (19.29, 87.47)	32.32 (11.43, 56.61)	20.25	38.52%	23	33.73%
17	11.94 (0.98, 27.47)	8.53 (0.99, 19.43)	3.41	28.56%	8.05	30.39%
18	99.76 (39.55, 162.58)	67.24 (27.6, 105.72)	32.52	32.60%	44.91	36.50%
19	34.46 (8.17, 68.51)	25.79 (6.32, 51.75)	8.67	25.16%	14.91	24.71%
20	11.72 (0.76, 27.61)	8.68 (0.76, 20.94)	3.04	25.94%	6.67	24.84%
21	103.13 (44.15, 166.81)	70.32 (30.77, 115.58)	32.81	31.81%	37.85	30.86%
22	86.67 (34.15, 146.83)	57.7 (23.63, 98.24)	28.97	33.43%	38.07	33.79%
23	36.88 (9.24, 73.7)	25.12 (5.48, 51.13)	11.76	31.89%	18.81	29.18%
24	193.69 (108.37, 279.41)	140.41 (74.09, 199.29)	53.28	27.51%	45.84	26.80%
25	116.54 (39.74, 193.36)	83.5 (28.22, 140.42)	33.04	28.35%	41.42	26.96%
26	232.29 (175.19, 287.57)	181.66 (171.6, 198.37)	50.63	21.80%	85.61	76.18%
**27**	**210.12 (161.63, 263.51)**	**166.64 (152.41, 182.25)**	**43.48**	**20.69%**	**72.04**	**70.71%**
**28**	**195.13 (150.86, 244.03)**	**159.8 (150.8, 174)**	**35.33**	**18.11%**	**69.97**	**75.10%**
29	125.64 (97.81, 162.23)	112.52 (96.01, 132.04)	13.12	10.44%	28.39	44.07%
30	77.95 (39.91, 118.25)	64.9 (29.13, 102.12)	13.05	16.74%	5.35	6.83%
31	52.75 (21.93, 86.86)	42.19 (14.59, 73.7)	10.56	20.02%	5.82	8.96%
32	31.95 (8.72, 60.79)	24.54 (6.15, 50.45)	7.41	23.19%	7.77	14.92%
33	43.73 (13.62, 78.98)	33.88 (8.51, 65.7)	9.85	22.52%	8.17	12.50%
**34**	**108.64 (93.5, 136.7)**	**101.07 (93.5, 115.08)**	**7.57**	**6.97%**	**21.62**	**50.05%**
**35**	**166.78 (114.26, 225.5)**	**113.73 (99.6, 137.84)**	**53.05**	**31.81%**	**73**	**65.62%**
36	124.98 (76.09, 176.54)	81.41 (54.42, 107.18)	43.57	34.86%	47.69	47.48%
**37**	**100.94 (57.89, 148.21)**	**61.26 (40.59, 81.49)**	**39.68**	**39.31%**	**49.42**	**54.72%**
38	75.3 (33.76, 117.91)	45.25 (22.69, 69.05)	30.05	39.91%	37.79	44.91%
39	44.45 (13.66, 78.45)	28.41 (10.7, 48.58)	16.04	36.09%	26.91	41.53%
40	46.85 (6.09, 107)	32.69 (4.51, 71.9)	14.16	30.22%	33.52	33.22%
41	12.57 (1.11, 31.14)	9.75 (0.69, 24.89)	2.82	22.43%	5.83	19.41%
42	232.02 (158.91, 312.28)	182.41 (123.61, 237.76)	49.61	21.38%	39.22	25.57%
43	196.12 (117.11, 273.54)	152.08 (88.27, 209.5)	44.04	22.46%	35.2	22.50%
44	80.65 (26.24, 146.36)	58.04 (19.79, 107.64)	22.61	28.03%	32.27	26.86%
45	140.37 (61.13, 222.16)	104.15 (37.88, 166.97)	36.22	25.80%	31.94	19.83%
46	30.18 (5.58, 65.73)	22.33 (3.71, 50.52)	7.85	26.01%	13.34	22.18%

Nodes in bold were employed as calibrations in the divergence time analyses. The mean node age and lower and upper bounds of the 95% HPD are shown for each node. Summary statistics provided include the absolute and percentage difference in mean node age (Dmean and%Dmean, respectively), and the absolute and percentage difference in the width of the 95% HPD Node Age (DHPD and%DHPD, respectively).

### The Effects of Fossil Preservation Rates on the Shape of Informative Node Age Prior Distributions

**Figure 5:**
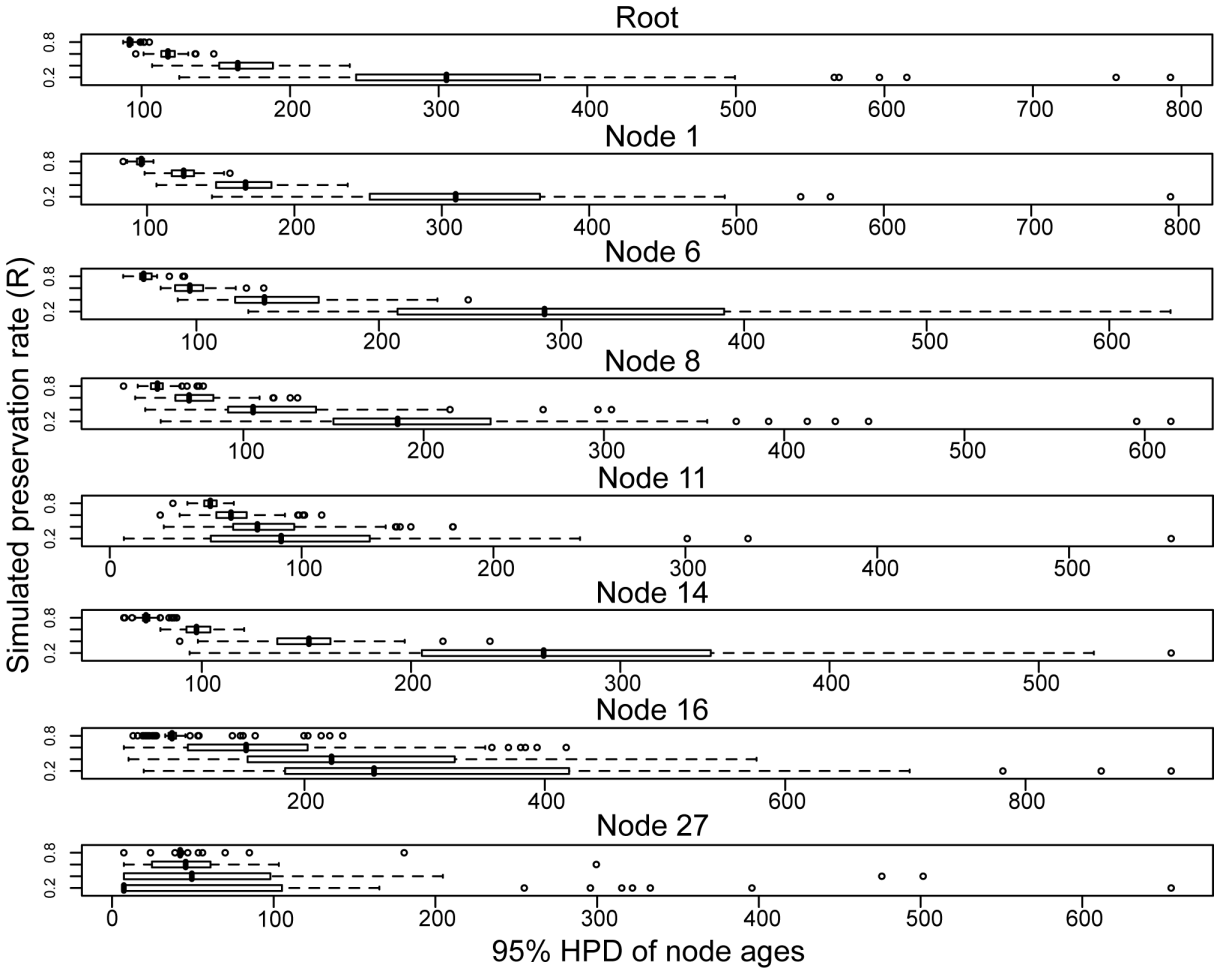
Simulating the impacts of incomplete preservation on the estimation of informative node age priors. To test the sensitivity of our method of prior estimation to the quality of the fossil record (i.e. under varying rates of fossil preservation), we simulated fossil occurrences for all fossil lineages in each of the eight constraint nodes and sub-sampled these under four preservation rates (0.2, 0.4, 0.6, 0.8). We constructed node age priors for each simulated data set, and summarized the results using boxplots of the 95% density of the estimated gamma distributions (measured in millions of years) for each of the four preservation rates grouped by calibration node (following the node numbering scheme in Figure 2, and Tables 1 and 2). Note that higher rates of fossil preservation reduce the 95% density of the gamma distribution significantly, which shows that when provided with data of higher quality (i.e. more meaningful for calibrating the age of the node in question), the method provides a more informative prior distribution. Conversely, when the method is provided with less informative fossil data (i.e. data simulated under a poor preservation rate), it provides a prior distribution that is less informative, and thus likely to have less of an impact in the resulting divergence time analysis.

The record of fossil echinoid genera is relatively complete for most clades, and this is likely due to a relatively high preservation rate throughout the history of this group [38], [44]. This characteristic of the echinoid fossil record provides an opportunity to examine the effects of incomplete preservation on the precision of informative node age priors estimated with our method. To test the sensitivity of our method, we simulated fossil occurrence data for the eight calibration nodes in the echinoid data set and sub-sampled these data under four preservation rates (i.e. 0.2, 0.4, 0.6, 0.8, respectively) and estimated informative node age priors. The results are shown in Figure 5. An obvious pattern in the results from all calibration nodes is that higher rates of fossil preservation (i.e. a more complete fossil record) reduce the 95% density of estimated gamma distributions significantly (see Figure 5), and this result suggests that when provided with data of higher quality (i.e. more meaningful for calibrating the age of the node in question), our method provides a more informative prior distribution. Conversely, when our method is provided with less useful fossil data (i.e. data simulated under a poor preservation rate), it provides a prior distribution that is less informative. Furthermore, aside from Node 27, data simulated under poor preservation rates have a consistently older gamma prior mean (results not shown). Despite this, the potential for bias in the resulting divergence time estimates may in reality be small, because this older mean is generally accompanied by a considerably more diffuse gamma distribution (i.e. a larger 95% density).

## Discussion

The method we present here employs analyses of paleobiological data to inform the construction of prior probability distributions on node ages in Bayesian divergence time analyses. Given the importance of the prior in Bayesian statistical inference generally [48], and molecular divergence time estimation specifically [15], we feel that this approach is philosophically attractive and likely to improve both the precision and accuracy of divergence time estimates. Our method provides a simple way of synthesizing data from the diverse fields of paleobiology and systematic biology, providing a foundation for increased accuracy and precision in dating lineage divergence events in the tree of life.

Our method makes several assumptions regarding both the appropriateness of the model and nature of the data. These assumptions include: 1) the origination (birth) and extinction (death) parameters of the branching model and the rate of preservation are constant through time; 2) all fossil lineages can be confidently assigned to the clade of interest as defined by the presence of well preserved morphological synapomorphies; 3) the stratigraphic ranges of fossil lineages are accurate both in terms of the appropriateness of the binning scheme employed and the absolute ages of the stratigraphic bins in question. Assumptions regarding the model and data are not explicitly accounted for in the uncertainty of prior distribution estimates, but these assumptions are not unique to our method and are in fact common to all molecular divergence time analyses that rely on fossil data for temporal calibration. Thus, while we feel that these assumptions likely impact the accuracy of the results, it is unclear how these issues can be accounted for in the current implementation of the method.

One input requirement for our method that is not required for other currently available divergence time analyses is that of a preservation rate estimate. As the results shown in Figure 2B show clearly, this parameter can have a large impact on the shape of the node age prior estimated for a given clade. Several analytical methods are currently available to estimate suitable preservation rates for a given clade using fossil range data similar to that required as input for our method. A simple approach to estimating preservation rates was developed by Foote and Raup called the range-frequency ratio method, or FreqRat [49]. This method relies on the assumption that under a simple model of cladogenesis in which the origination rate is not dramatically greater than the extinction rate the true distribution of fossil ranges should be exponential. The random process of fossil preservation and recovery will thus tend to degrade this distribution of fossil ranges yielding a distribution that is enriched for singletons (i.e. fossil taxa confined to a single stratigraphic interval). The FreqRat method thus uses the assumed degradation of fossil ranges to estimate the preservation rate that produced the observed distribution of fossil ranges for the clade of interest [49]. The simplicity of this approach is appealing, and we provide the option of automatically estimating preservation rates using the FreqRat method in the SNAPE v1.0 software. But it is important to note here that in our experience the preservation rate estimates provided by FreqRat are at times unrealistic, and thus this approach should be used with considerable caution. A second approach would be to apply Alroy's [42], [50] two-timer rates method, which provides estimates of the preservation rate based on the ratio of different fossil range classes. There are many approaches for estimating preservation rates available (see for example [51]), and ultimately the users of this method will need to decide which approach is most appropriate for their taxonomic group of interest.

Given our method's reliance on quality fossil range data, it is possible that its primary utility will be realized by those researchers interested in estimating divergence times in groups with relatively large and diverse fossil records. This includes groups whose habitat preferences place them in convenient proximity to suitable depositional environments for fossil preservation (e.g. eutrophic lakes, marine intertidal zones, etc.), or groups whose anatomy provides a wealth of readily fossilized parts that retain a suitable number of taxonomically useful characteristics (e.g. foraminifera, arthropods, angiosperm pollen, etc.). Additionally, we feel that this method holds great promise in estimating informative node age priors for relatively deep divergences in the tree of life, particularly those in which the fossil record may be relatively poor near the presumed MRCA, but relatively rich later in their history (e.g. angiosperms, mammals, primates, and birds). The current implementation of our method is not applicable to divergence time studies utilizing temporal information from a single fossil lineage, despite the fact that these studies are arguably most in need of a method to quantify calibration uncertainty. The precise number of fossil lineages required to estimate priors that are more informative than simple minimum age constraints is dependent on too many parameters to confidently estimate. The fundamental limitation to the application of our method in clades with very few fossil lineages lies in the ability to estimate origination and extinction rate parameters, as this is highly contingent on the number of stratigraphic intervals in question and the amount of stratigraphic overlap between fossil ranges. While the theoretical foundations of our method could be applied to clades with one fossil lineage or even a complete absence of fossil lineages, the key parameters of the absolute lineage origination and extinction rates would need to be estimated in some way, and such an estimate will be accompanied by significant error.

It is now well known that Bayesian molecular divergence time methods such as BEAST can yield results that are inconsistent with calibration prior densities when provided with multiple fossil calibrations [15], [31], [52]. The issue arises due to the conflict between the node age suggested by the prior distribution and the reality that descendant nodes must be younger or maximally the same age as ancestral nodes deeper in the tree. To identify the potential impact of prior truncation Heled and Drummond [46] suggest that users of the BEAST software package perform an analysis of their data without any data to identify any inconsistencies between the user-defined node age prior distributions and the joint prior distribution resulting from the combined effects of all of the node age priors and topological constraints. The results of our analyses of echinoid divergence times provide an important perspective on the truncation of joint priors because we performed identical analyses using both minimum-age (uniform) priors and informative gamma-distributed priors. When considering just prior truncation on the upper bound or 95% density of the eight calibration priors, we found that the average truncation was 99 million years for minimum age priors and 16 million years for gamma-distributed priors (Table 1). This dramatic difference in prior truncation points to the inadequacy of minimum age priors in divergence time estimation and highlights the importance of the node age prior paramter choice.

The approach to estimating node age priors that we present here is computationally simple, powerful, and sufficiently flexible to be used in a diversity of taxonomic groups. Future work could improve upon our approach by developing an iterative framework for the estimation of multiple calibrations in a clade, or perhaps our method could be integrated into a Bayesian divergence time software package directly, thus removing the need for fitting a parametric distribution entirely. Given that this is a field of active development, the years to come are sure to see important advances in establishing objective means of estimating node age priors for dating divergence events in the tree of life.

## Supporting Information

Figure S1 **The highest** ***a posteriori*** **chronogram for the echinoid BEAST analyses performed with informative gamma-distributed calibration priors.** The clade credibility values are shown above the branches.
(PDF)

Figure S2 **Joint prior tree estimated with echinoid data using uniformly distributed minimum node age priors.** Node bars show the 95% HPD of node height.
(PDF)

Figure S3 **Joint prior tree estimated with echinoid data using gamma-distributed informative node age priors.** Node bars show the 95% HPD of node height.
(PDF)

Table S1 **The table shows a comprehensive listing of fossil echinoid genera used in our analysis.** Fossil taxa are organized in groups representing the calibration node (i.e. clade) to which these fossil lineages are associated.
(XLS)

File S1 **R scripts used to construct the simulated data sets employed to test the method.**
(R) Click here for additional data file.

File S2 **Raw fossil occurrence data used to construct the example prior distribution for the MRCA of Rodentia presented in** **Figure 2B** **.**
(CSV)
