# Supplementary figures and images for: A Simple Method for Estimating Informative Node Age Priors for the Fossil Calibration of Molecular Divergence Time Analyses
Source: PLoS One. 2013 Jun 5;8(6):e66245. doi: 10.1371/journal.pone.0066245 (PMC3673923; doi:10.1371/journal.pone.0066245)

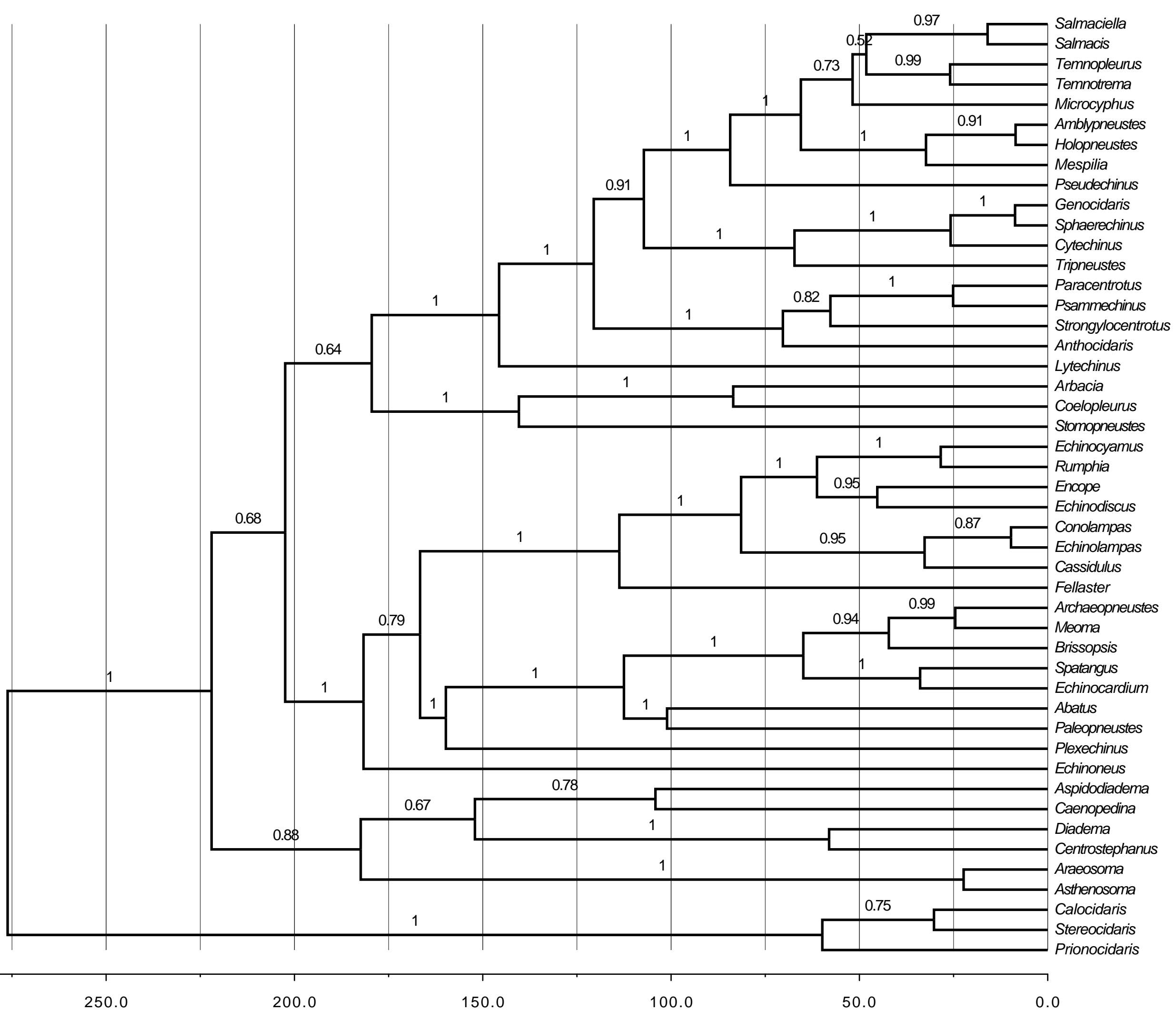

Supplement: Figure S1 — The highest a posteriori chronogram for the echinoid BEAST analyses performed with informative gamma-distributed calibration priors. The clade credibility values are shown above the branches. (PDF) [file pone.0066245.s001.pdf]

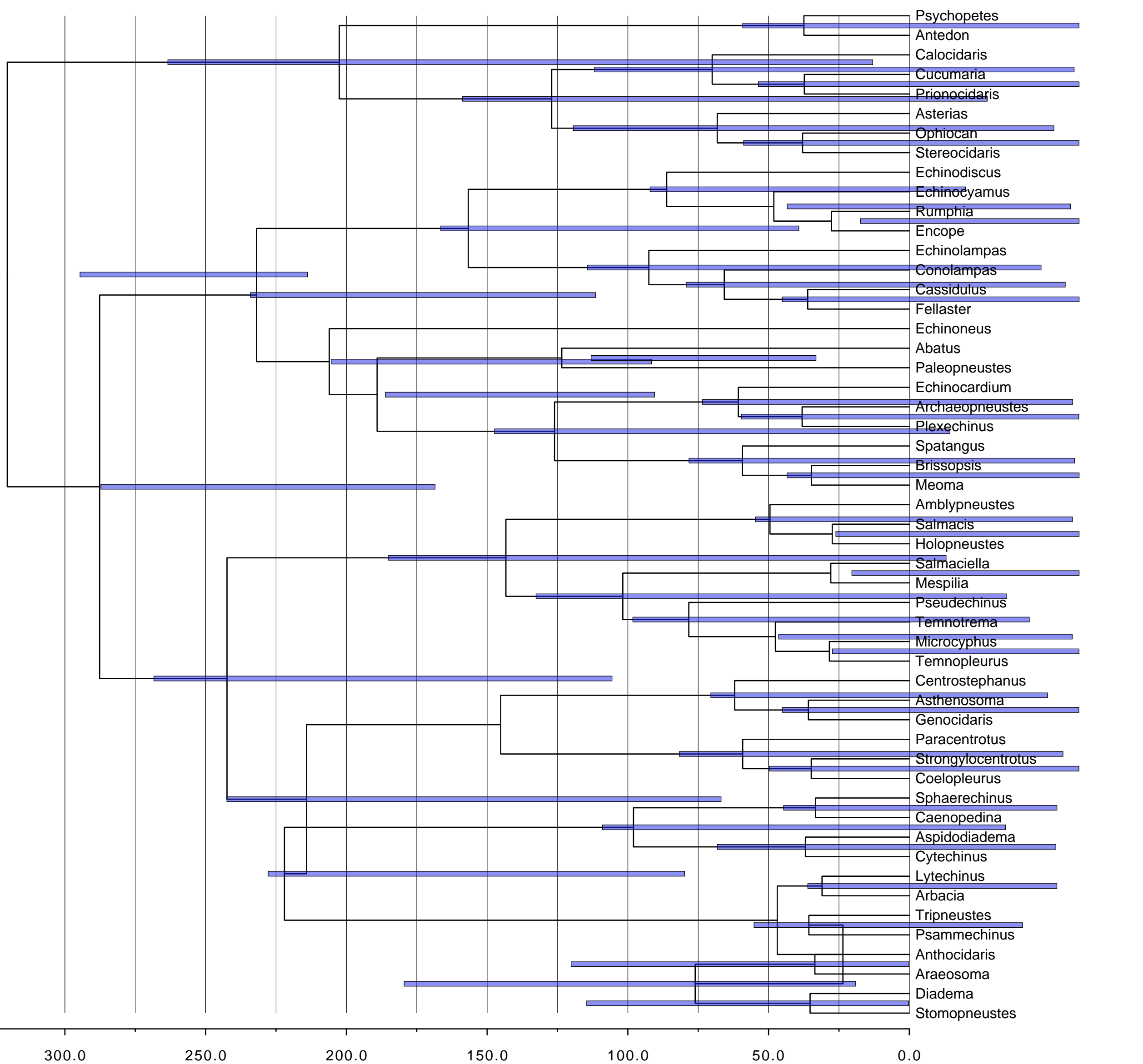

Supplement: Figure S2 — Joint prior tree estimated with echinoid data using uniformly distributed minimum node age priors. Node bars show the 95% HPD of node height. (PDF) [file pone.0066245.s002.pdf]

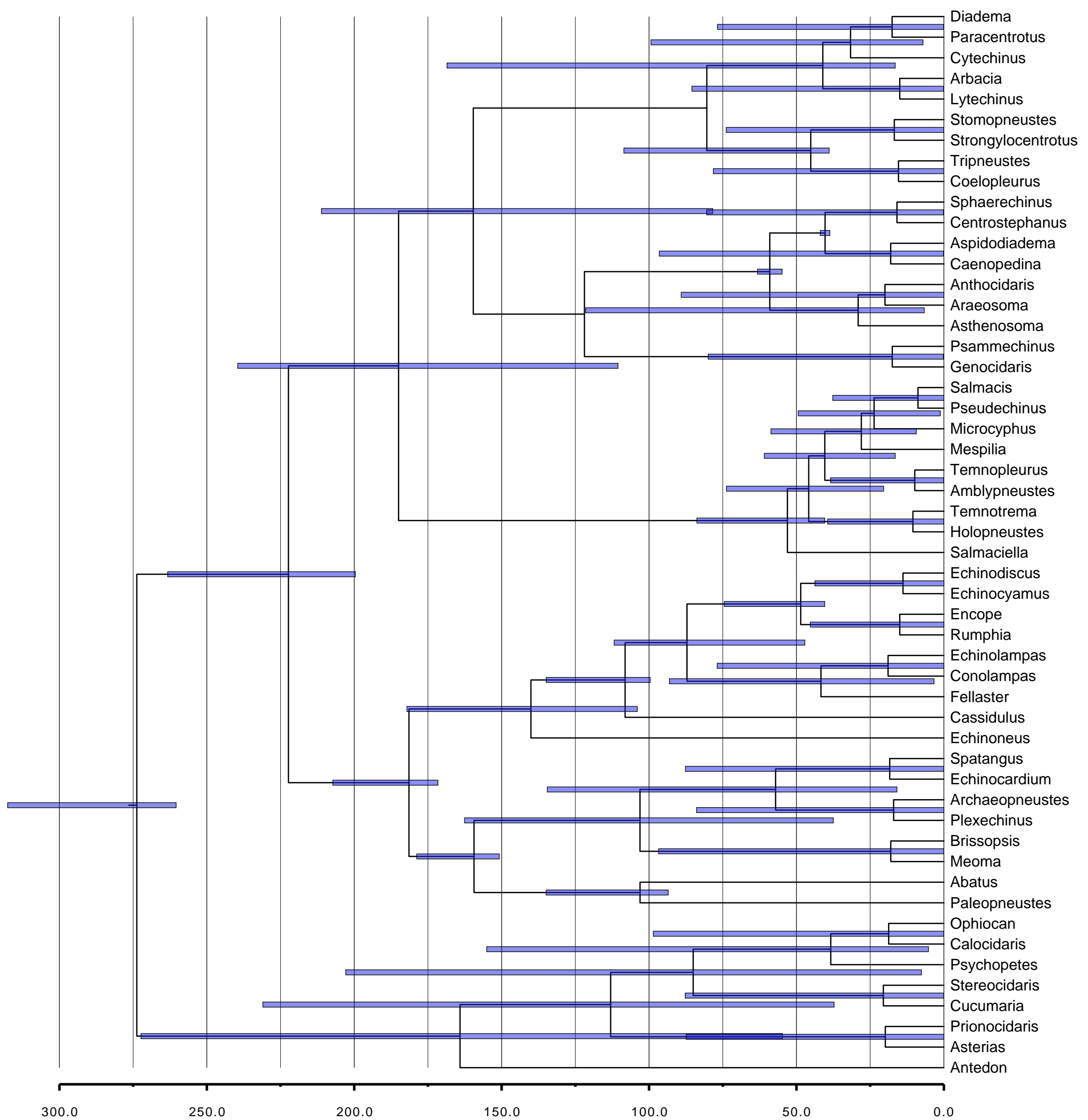

Supplement: Figure S3 — Joint prior tree estimated with echinoid data using gamma-distributed informative node age priors. Node bars show the 95% HPD of node height. (PDF) [file pone.0066245.s003.pdf]
